# Alcohol as a reported frequent problem and sociodemographic correlates in a Waorani community in the Ecuadorian Amazon: a community-based cross-sectional census study

**DOI:** 10.3389/fpubh.2026.1898589

**Published:** 2026-10-19

**Authors:** Raynier Zambrano-Villacres, Franklin Roberto Quishpi-Choto, Erika Gabriela Quishpi-Contento, Jaen Cagua-Ordoñez, Nelly Andrade-Mejia, Daniel Simancas-Racines

**Affiliations:** 1 Facultad de Ciencias de la Salud y Desarrollo Humano, Universidad ECOTEC, Samborondón, Ecuador; 2 Escuela de Posgrado, Doctorado en Ciencias Biomédicas, Instituto Universitario Italiano de Rosario (IUNIR), Rosario, Argentina; 3 Departamento de Geografía, Universitat Rovira i Virgili, Programa Doctoral Ciudad, Territorio y Planificación Sostenible, Tarragona, Spain; 4 Facultad de Ciencias de la Salud y Bienestar Humano, Universidad Tecnológica Indoamérica, Quito, Ecuador; 5 Facultad de Ciencias de la Salud, Universidad de Especialidades Espíritu Santo (UEES), Samborondón, Ecuador; 6 Facultad de Ciencias de la Salud y Bienestar Humano, Universidad Tecnológica Indoamérica, Ambato, Ecuador

**Keywords:** alcohol, community health, cross-sectional studies, Ecuadorian Amazon, indigenous health, social determinants of health, Waorani

## Abstract

**Background:**

Alcohol-related concerns among Indigenous populations should be interpreted within their specific social, territorial, and historical contexts. Quantitative evidence from Waorani communities in the Ecuadorian Amazon remains limited. This study estimated the proportion of census records in which alcohol was identified as a frequent problem and examined associated sociodemographic characteristics in the Nuevo Amanecer Waorani community.

**Methods:**

We conducted a community-based cross-sectional secondary analysis of 125 individual census records from Nuevo Amanecer, Shell Parish, Pastaza Province, Ecuador. The primary outcome was derived from the census item “frequent problem” and was classified as positive when alcohol was selected. Crude and adjusted prevalence ratios (cPRs and aPRs) were estimated using Poisson regression with a log link and HC3 robust sandwich variance. The primary adjusted model included sex, age group, and partnership status. Sensitivity analyses were restricted to adults aged ≥18 years and additionally evaluated educational attainment.

**Results:**

Alcohol was selected as the frequent problem in 81 of 125 census records (64.8%, 95% CI 56.1–72.6). The primary complete-case model included 123 records. Male sex was associated with a higher prevalence of the outcome (aPR 1.31; 95% CI 1.03–1.67). Compared with individuals aged <18 years, higher adjusted prevalence ratios were observed for ages 18–24 years (aPR 2.64; 95% CI 1.58–4.42), 25–34 years (aPR 2.64; 95% CI 1.57–4.42), and ≥45 years (aPR 2.11; 95% CI 1.16–3.83). Stable partnership status showed a more modest association (aPR 1.30; 95% CI 1.00–1.68). In the adult-only analysis (*n* = 80), the association with male sex persisted (aPR 1.24; 95% CI 1.01–1.53), whereas the age pattern changed and the estimate for stable partnership attenuated (aPR 1.21; 95% CI 0.97–1.51).

**Conclusion:**

Alcohol was selected as the frequent problem in nearly two-thirds of census records in this Waorani community. Male sex was the most consistent sociodemographic correlate across analyses, whereas age and partnership associations were more sensitive to the analytical population. These findings should not be interpreted as estimates of individual alcohol consumption, hazardous drinking, or alcohol use disorder. Further research should incorporate culturally and linguistically appropriate, validated alcohol-specific measures, respondent and household identifiers, and longitudinal or mixed-methods approaches developed in collaboration with Waorani leadership.

## Introduction

Alcohol-related harms constitute a major global public health concern and contribute substantially to morbidity, mortality, disability, and social burden worldwide (1). Among Indigenous populations, substance use has also been examined in relation to historical and lived trauma, including experiences of cultural loss and disruption (2). These patterns should not be interpreted as characteristics inherent to Indigenous identity; rather, they underscore the importance of considering the historical, structural, and social contexts in which alcohol-related concerns occur.

Among Indigenous peoples in Colombia, qualitative evidence has described alcohol consumption as embedded within complex social and cultural processes involving interactions between Indigenous and non-Indigenous systems, family and community authority, education, spirituality, and processes of sociocultural change (3). Quantitative evidence is also limited and heterogeneous. In a Colombian Indigenous population living in Bogotá, problem drinking measured using the Alcohol Use Disorders Identification Test (AUDIT) was associated with male sex and longer residence in the city (4). These findings illustrate the potential relevance of sociodemographic and contextual characteristics but should not be generalized across Indigenous populations or settings.

Evidence regarding alcohol-related concerns specifically among Waorani communities remains limited. More broadly, research on substance-use responses in Indigenous populations has emphasized the potential value of culturally tailored and community-based approaches rather than assuming that interventions developed for other populations can be transferred without adaptation (5). This consideration is particularly relevant in Waorani research, where previous work in Ecuador has highlighted the importance of transparent communication, meaningful consent, respect for Indigenous governance, and collaboration with community members (6). Family-based approaches have also been evaluated as a potential component of alcohol-harm reduction in Indigenous communities, although the available evidence remains limited (7). Together, these considerations reinforce the need for context-specific epidemiological evidence before developing or implementing prevention strategies in individual Waorani communities.

Therefore, this study aimed to estimate the proportion of census records in which alcohol was identified as a frequent problem and to examine its sociodemographic correlates among members of the Nuevo Amanecer Waorani community in Shell Parish, Pastaza Province, Ecuador. Specifically, we evaluated differences according to sex, age group, partnership status, and educational level using a community-based cross-sectional census approach.

## Methods

### Study design, setting, and population

We conducted a community-based cross-sectional secondary analysis of census data from the Nuevo Amanecer Waorani settlement, located in Shell Parish, Pastaza Province, Ecuador. Shell Parish is a peri-urban area near Puyo and includes Waorani families who have moved from more traditional Amazonian territories. The study aimed to estimate the proportion of census records in which alcohol was identified as a frequent problem and to examine its sociodemographic correlates.

The analytical unit was the individual census record. The source database contained 125 records corresponding to children, adolescents, and adults from the Nuevo Amanecer community. Because the original census was conducted using a household-based approach, the individual represented in each record was not necessarily the person who directly provided the information.

All 125 records with available information on the frequent-problem item were included in the descriptive analysis. The primary multivariable analysis used complete cases for the outcome, sex, age group, and partnership status, resulting in an analytical sample of 123 records. Because respondent modality was not recorded and proxy reporting was more likely to affect younger community members, a sensitivity analysis was additionally restricted to records corresponding to individuals aged 18 years or older.

The available analytical dataset did not contain a separate denominator of eligible residents who were not included in the census; consequently, a conventional participation or response rate could not be independently calculated from the analytical file.

### Census instrument, data collection, and variables

Data were obtained using a structured community census form covering sociodemographic, household, educational, health, and social-development information. The original community census form is provided as Supplementary File S1.

Within the health and development section, the relevant item was worded: “PROBLEMÁTICA FRECUENTE: 1. ALCOHOL, 2. DROGA, 3. SECTAS, 4. OTRA ¿CUÁL?” The source database additionally used code 0 for “Ninguno” (none). Accordingly, the available categories were none, alcohol, drugs, sects, and other.

For the primary outcome, records were classified as outcome-positive when alcohol was selected and as outcome-negative when none or another non-alcohol category was recorded. This variable was not interpreted as a measure of individual alcohol consumption, frequency or quantity of drinking, episodic heavy drinking, hazardous alcohol use, alcohol use disorder, or age of alcohol initiation. The outcome therefore represents whether alcohol was identified as the frequent problem in the census record.

Census information was obtained primarily from the household head through interviewer-administered data collection. Individuals aged 15 years or older could provide responses directly to selected questions when present during data collection. For minors aged 15–17 years who participated directly, parental authorization and informed assent from the minor were obtained. For individuals younger than 15 years, information was provided by the household head. However, the analytical database did not retain a variable identifying whether each individual record reflected direct or proxy reporting; direct and proxy responses could therefore not be distinguished systematically at the record level.

The present study represents a secondary analysis of an existing community census. Thus, the interviewer-administered household approach formed part of the original census procedures rather than a data-collection strategy selected specifically for the present alcohol-related analysis. No additional interviews, self-administered questionnaires, or alcohol-specific screening instruments were administered for this secondary analysis. The original census was designed for broader community characterization rather than for clinical assessment or screening of alcohol consumption; consequently, the available frequent-problem item should not be interpreted as an alcohol screening instrument.

The primary explanatory variables were sex, age group, and partnership status. Sex was classified as female or male when a valid source code was available. One record contained a non-classifiable sex code and was retained in descriptive analyses but treated as missing in regression models requiring sex.

Age was categorized as <18, 18–24, 25–34, 35–44, and ≥45 years. Age was modeled categorically because the descriptive pattern was not consistent with a single linear association and because this specification allowed age-group-specific prevalence ratios to be estimated without imposing a constant effect across the age range.

Original marital status comprised married, free union, single parent, and single categories. For the primary analysis, married individuals and those in free union were classified as having a stable partner, whereas single and single-parent individuals were classified as not having a stable partner. One record with missing marital-status information was not imputed.

Educational attainment was summarized using the documented categories of primary/basic education, secondary/high school, technical education, and university education. Three records contained source code 0, which could not be mapped to a documented educational category and were therefore retained as unclassified/missing. Educational attainment was evaluated in an additional sensitivity analysis using the documented ordered categories.

Other available sociodemographic and health characteristics were summarized descriptively but were not automatically entered into the adjusted model. Covariates for the primary model were selected according to the study objective and their conceptual relevance rather than according to statistical significance in bivariate analyses.

Data were reconstructed directly from the original coded census spreadsheet. Variable labels and coding were standardized, and consistency checks were performed for age, sex, marital status, educational level, and the frequent-problem item. Values that could not be mapped unambiguously to a documented category were retained as missing or unclassified rather than reassigned. No statistical imputation was performed.

### Statistical analysis

Continuous variables were summarized using means and standard deviations, and categorical variables using absolute and relative frequencies. The primary descriptive estimate was the proportion of census records in which alcohol was identified as the frequent problem. Exact numerators and denominators were reported, and 95% confidence intervals for proportions were calculated using the Wilson method. The original distribution of the frequent-problem item was also summarized to provide transparency regarding construction of the binary outcome.

Associations between sociodemographic characteristics and the binary outcome were estimated using generalized linear models with a Poisson distribution and log link. Because the outcome was common, prevalence ratios were preferred to odds ratios as the primary measure of association. Robust sandwich variance estimators were used to account for misspecification of the conventional Poisson variance for a binary outcome. HC3 covariance estimates were used for the primary analysis because of the modest analytical sample and their greater protection against leverage-related small-sample bias.

Crude prevalence ratios (cPRs) with 95% confidence intervals were first estimated separately for sex, age group, and partnership status. The primary multivariable model simultaneously included sex, age group, and partnership status and reported adjusted prevalence ratios (aPRs), 95% confidence intervals, and two-sided *p* values. Female sex, age <18 years, and absence of a stable partner were used as reference categories. Covariates were selected according to the study objective and conceptual relevance and were not chosen on the basis of statistical significance in univariable analyses.

The primary regression analysis used complete cases for the outcome, sex, age group, and partnership status. No missing-data imputation was performed. Because respondent modality was not retained at the individual-record level and records corresponding to participants younger than 15 years necessarily relied on household-head reporting, while those aged 15–17 years could reflect either direct or proxy responses, a sensitivity analysis was restricted to individuals aged ≥18 years.

A second sensitivity analysis additionally incorporated educational attainment using only documented education categories with an interpretable ordinal ordering. Records with an undocumented education code were excluded from this sensitivity model rather than reassigned to an unsupported category. This analysis was used to assess whether adjustment for educational attainment materially altered the associations observed in the primary model.

Model adequacy was assessed by evaluating convergence, parameter stability, multicollinearity, influential observations, and sensitivity to the robust covariance specification. Generalized variance inflation factors were used to assess multicollinearity. Influence diagnostics included Cook’s distance and leave-one-out refitting of the primary model. As an additional variance-sensitivity analysis, estimates obtained using HC3 covariance were compared with HC0 and HC1 robust covariance estimators. These analyses were considered robustness checks rather than additional hypothesis-generating models.

Because Poisson regression was used as a working mean model for a binary outcome, conventional Poisson dispersion assumptions were not used as a criterion for accepting or rejecting the model. Inference relied on robust sandwich variance estimation.

The data originated from a community census rather than a probability sample. Accordingly, descriptive proportions characterize the enumerated census records. Confidence intervals and regression-based uncertainty estimates were used to quantify statistical precision and to support a model-based interpretation of associations beyond the observed records, rather than to represent conventional sampling error within the enumerated census itself.

A unique household identifier was not available in the analytical database; therefore, within-household correlation could not be explicitly modeled using household-clustered variance estimators. This limitation was considered when interpreting statistical precision.

All analyses were conducted in R using reproducible scripts. Statistical interpretation emphasized effect estimates and 95% confidence intervals rather than statistical significance alone. A two-sided *p* value <0.05 was used as a conventional reference for the primary analysis.

### Ethical considerations

The study protocol was reviewed and approved by the Research Ethics Committee on Human Beings of the National University of Chimborazo (CEISH-UNACH; approval code: 04-0000-FRQC-07-02-2025). Prior to field activities, authorization was also obtained from Waorani community leadership in accordance with principles of community governance, collective participation, and respect for Indigenous self-determination.

Participation was voluntary. Census information was collected primarily from household heads. Individuals aged 15 years or older could respond directly to selected questions. When minors aged 15–17 years participated directly, parental authorization and informed assent from the minor were obtained. Information for individuals younger than 15 years was provided by the household head. Data were recorded using numerical identifiers for analytical purposes, and results were reported only in aggregate form to reduce risks of identification and stigmatization within this small Indigenous community.

## Results

### Census characteristics and distribution of the frequent-problem item

The source census database comprised 125 individual records from the Nuevo Amanecer Waorani community. The mean age was 25.1 years (SD 13.9), and 45 records (36.0%) corresponded to individuals aged <18 years. Sex was recorded as female in 62 records (49.6%) and male in 62 (49.6%); one record (0.8%) contained a non-classifiable source code. The main sociodemographic characteristics of the census population are summarized in Table 1.

**Table 1:** Sociodemographic characteristics of the Nuevo Amanecer Waorani census records (*N* = 125).

Characteristic	*n* (%) or mean (SD)
Age, years	25.1 (13.9)
Age group	
<18 years	45 (36.0)
18–24 years	23 (18.4)
25–34 years	19 (15.2)
35–44 years	26 (20.8)
≥45 years	12 (9.6)
Sex	
Female	62 (49.6)
Male	62 (49.6)
Missing/non-classifiable	1 (0.8)
Literacy	
Yes	111 (88.8)
No	14 (11.2)
Religion	
Catholic	112 (89.6)
Evangelical	13 (10.4)
Partnership status	
No stable partner	68 (54.4)
Stable partner	56 (44.8)
Missing	1 (0.8)
Educational level	
Primary/basic	53 (42.4)
Secondary/high school	53 (42.4)
Technical	8 (6.4)
University	8 (6.4)
Missing/unclassified	3 (2.4)
Health status	
Good	112 (89.6)
Regular	13 (10.4)

Data are presented as *n* (%) unless otherwise indicated. Percentages use the complete census denominator (*N* = 125), with missing or non-classifiable source values reported explicitly.

Alcohol was selected as the frequent problem in 81 of 125 census records (64.8%, 95% CI 56.1–72.6). Among the remaining records, 43 (34.4%) were classified as none and one (0.8%) as other; no records were classified as drugs or sects. No values were missing for the frequent-problem item.

The proportion of census records in which alcohol was selected as the frequent problem varied substantially across sociodemographic groups (Figure 1). Among records with classifiable sex, alcohol was selected in 48 of 62 male records (77.4%, 95% CI 65.6–86.0) and 33 of 62 female records (53.2%, 95% CI 41.0–65.1).

**Figure 1:**
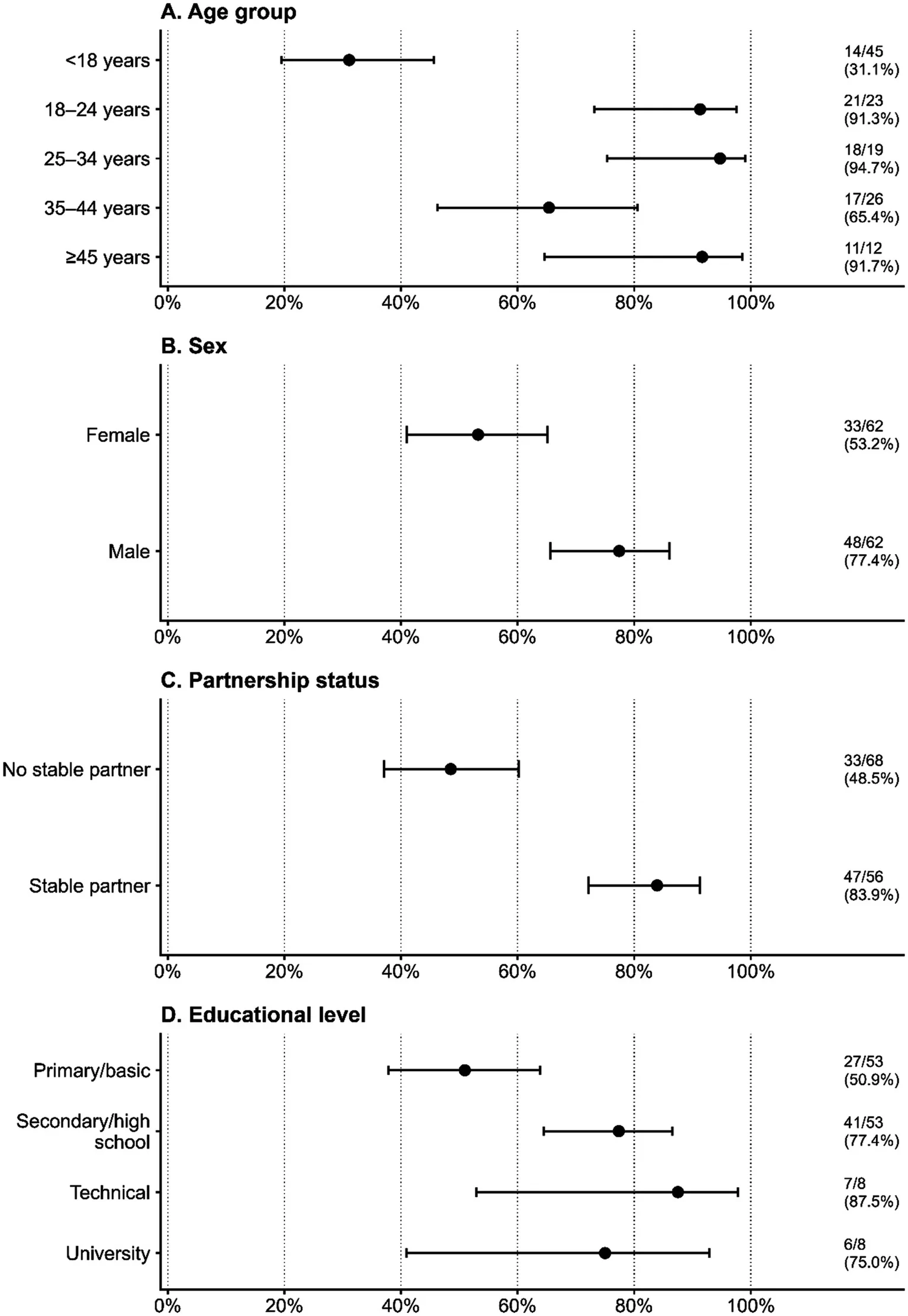
Proportion of census records in which alcohol was selected as the frequent problem according to sociodemographic characteristics. Points represent observed proportions and horizontal lines 95% Wilson confidence intervals. Labels indicate *n/N* (%).

Marked heterogeneity was observed across age groups. Alcohol was selected in 14 of 45 records corresponding to individuals aged <18 years (31.1%; 95% CI 19.5–45.7), compared with 21 of 23 records among those aged 18–24 years (91.3%; 95% CI 73.2–97.6), 18 of 19 among those aged 25–34 years (94.7%; 95% CI 75.4–99.1), 17 of 26 among those aged 35–44 years (65.4%; 95% CI 46.2–80.6), and 11 of 12 among those aged ≥45 years (91.7%; 95% CI 64.6–98.5).

Differences were also observed according to partnership status. Alcohol was selected in 47 of 56 records corresponding to individuals with a stable partner (83.9%; 95% CI 72.2–91.3), compared with 33 of 68 records among those without a stable partner (48.5%; 95% CI 37.1–60.2). Across educational categories, the proportions were 50.9% (27/53) for primary/basic education, 77.4% (41/53) for secondary/high school education, 87.5% (7/8) for technical education, and 75.0% (6/8) for university education. Confidence intervals were wider for technical and university education because of the smaller numbers of records in these categories (Figure 1).

### Crude and adjusted sociodemographic associations

The primary complete-case regression analysis included 123 census records, of which 80 were classified as outcome-positive and 43 as outcome-negative. Two records were excluded because of missing or non-classifiable information in variables included in the primary adjustment set. Crude and adjusted prevalence ratios are presented in Table 2.

**Table 2:** Crude and adjusted prevalence ratios for alcohol being selected as the frequent problem in the primary complete-case analysis (*N* = 123).

Characteristic	Alcohol selected, *n/N* (%)	cPR (95% CI)	aPR (95% CI)	*p* value
Sex				
Female	33/62 (53.2)	1.00 (reference)	1.00 (reference)	
Male	47/61 (77.0)	1.45 (1.10–1.91)	1.31 (1.03–1.67)	0.027
Age group				
<18 years	13/43 (30.2)	1.00 (reference)	1.00 (reference)	
18–24 years	21/23 (91.3)	3.02 (1.86–4.90)	2.64 (1.58–4.42)	<0.001
25–34 years	18/19 (94.7)	3.13 (1.94–5.05)	2.64 (1.57–4.42)	<0.001
35–44 years	17/26 (65.4)	2.16 (1.25–3.74)	1.72 (0.94–3.16)	0.081
≥45 years	11/12 (91.7)	3.03 (1.84–5.00)	2.11 (1.16–3.83)	0.014
Partnership status				
No stable partner	33/67 (49.3)	1.00 (reference)	1.00 (reference)	
Stable partner	47/56 (83.9)	1.70 (1.30–2.24)	1.30 (1.00–1.68)	0.046

cPR, crude prevalence ratio; aPR, adjusted prevalence ratio; CI, confidence interval. Adjusted estimates include sex, age group, and partnership status and were obtained using modified Poisson regression with HC3 robust variance. Counts correspond to the complete-case analytical sample (*N* = 123).

In the adjusted model, the prevalence ratio for male compared with female records was 1.31 (95% CI 1.03–1.67; *p* = 0.027). Compared with records corresponding to individuals aged <18 years, the adjusted prevalence ratios were 2.64 (95% CI 1.58–4.42; *p* < 0.001) for those aged 18–24 years, 2.64 (95% CI 1.57–4.42; *p* < 0.001) for those aged 25–34 years, 1.72 (95% CI 0.94–3.16; *p* = 0.081) for those aged 35–44 years, and 2.11 (95% CI 1.16–3.83; *p* = 0.014) for those aged ≥45 years.

For records corresponding to individuals with a stable partner, the adjusted prevalence ratio relative to those without a stable partner was 1.30 (95% CI 1.00–1.68; *p* = 0.046). The proximity of the lower confidence limit to the null reflected the limited precision of this estimate.

### Sensitivity analyses

Because respondent identity was not recorded for individual census records and proxy reporting was more likely to affect younger age groups, the principal sensitivity analysis was restricted to records corresponding to individuals aged ≥18 years. This analysis included 80 records, of which 67 had alcohol selected as the frequent problem (83.8%; 95% CI 74.2–90.3).

In the adult-only adjusted model, the prevalence ratio for male compared with female records was 1.24 (95% CI 1.01–1.53; *p* = 0.042). Using ages 18–24 years as the reference category, the aPR was 1.01 (95% CI 0.84–1.20) for ages 25–34 years, 0.67 (95% CI 0.49–0.91) for ages 35–44 years, and 0.84 (95% CI 0.66–1.07) for ages ≥45 years. The adjusted prevalence ratio for stable partnership status was 1.21 (95% CI 0.97–1.51; *p* = 0.099). The complete adult-only estimates are displayed in Figure 2.

**Figure 2:**
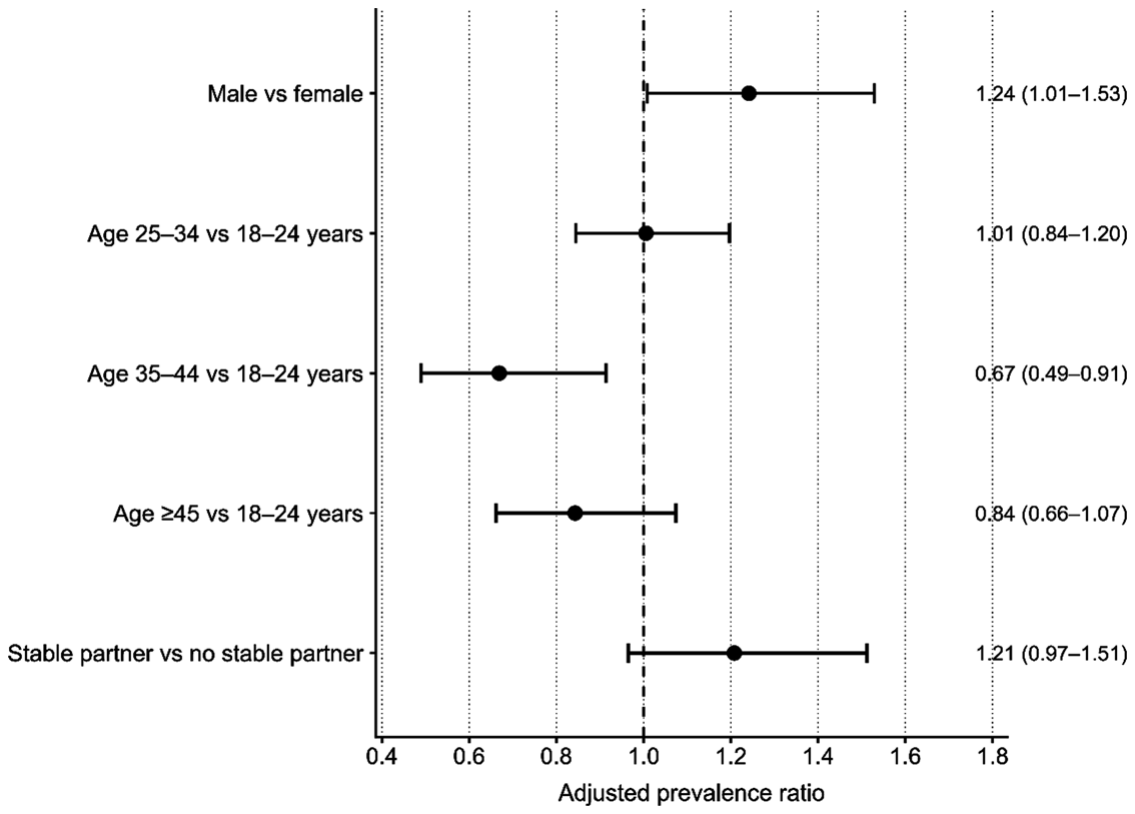
Adjusted prevalence ratios in the sensitivity analysis restricted to adults aged ≥18 years. Points show aPRs with 95% CIs from modified Poisson regression with HC3 robust variance. Reference categories were female sex, age 18–24 years, and no stable partner; the dashed line indicates aPR = 1.0.

A second sensitivity analysis incorporated educational attainment into the adjusted model using only documented educational categories with an interpretable ordinal ordering. Educational attainment was not clearly associated with the outcome (aPR per one-category increase, 0.91; 95% CI 0.80–1.04; *p* = 0.185). Inclusion of education produced only modest changes in the estimates for the main covariates; the aPR for male sex, for example, was 1.34 (95% CI 1.05–1.71).

The primary model converged successfully and showed no evidence of problematic multicollinearity or disproportionate influence from individual observations. Adjusted generalized variance inflation factors were approximately 1.05 for sex, 1.09 for age group, and 1.35 for partnership status, and the maximum Cook’s distance was approximately 0.014. Estimates were materially similar when HC0, HC1, and HC3 robust covariance specifications were compared. Leave-one-out refitting likewise showed limited sensitivity of the principal estimates to exclusion of individual census records. Model diagnostics and robustness analyses supported the primary model; detailed results are provided in Supplementary Table S2.

## Discussion

In this community-based cross-sectional census of the Nuevo Amanecer Waorani settlement, alcohol was identified as the frequent problem in nearly two-thirds of census records. The distribution of this outcome varied across sociodemographic groups. In the primary adjusted analysis, male sex, several adult age categories, and stable partnership status were associated with higher prevalence ratios, although the adult-only sensitivity analysis substantially changed the interpretation of the age and partnership findings.

Male sex was the most consistent sociodemographic correlate across analyses. In the complete-case model, records corresponding to males had an adjusted prevalence approximately 31% higher than those corresponding to females, and a similar association persisted after restricting the analysis to adults. This pattern is broadly consistent with previous studies reporting higher levels of alcohol use or alcohol-related problems among men in Indigenous and other populations (4, 8, 9). Potential explanations may involve gendered social roles, occupational and social contexts, and differences in exposure to alcohol-related environments, although these mechanisms were not measured in the present study (8–10). Importantly, alcohol was also identified as the frequent problem in more than half of female records, indicating that the issue should not be framed exclusively as a male phenomenon.

The age findings require greater caution. In the full analytical sample, several adult age groups showed substantially higher prevalence ratios than individuals aged <18 years. However, when the analysis was restricted to adults, the pattern changed considerably. Individuals aged 25–34 years had an estimate similar to those aged 18–24 years, whereas those aged 35–44 years showed a lower adjusted prevalence and the estimate for those aged ≥45 years was less precise. These findings suggest that the large age contrasts observed in the full model were driven in part by differences between minors and adults rather than by a consistent gradient across adulthood.

The present findings should not be interpreted as evidence of age at drinking initiation because the census did not measure this construct. Studies in other Indigenous youth populations have documented age-related differences in alcohol use, including earlier initiation in some settings (11), but such findings cannot be extrapolated directly to Nuevo Amanecer. Research among Indigenous youth has also linked alcohol-related behaviors with negative affect, school adjustment, caregiver monitoring, peer drinking, cultural identity, and parent–child communication (12–14). These studies illustrate the importance of age-specific social contexts but do not establish the mechanisms underlying the age differences observed in the present census.

The non-linear pattern across age categories also supports the decision to model age categorically rather than imposing a single linear effect. Interpretation is further complicated by the absence of information on respondent modality for individual records. Because census information was obtained primarily from household heads, lower reporting among records corresponding to minors may reflect true age-related differences, proxy reporting, or differences in how the census question was interpreted.

Stable partnership status also showed less consistent evidence after sensitivity analysis. In the primary model, records corresponding to individuals with a stable partner had a higher adjusted prevalence, but the lower confidence limit was close to the null. In the adult-only analysis, the estimate attenuated further and its confidence interval included 1.0. Partnership status should therefore be interpreted as a contextual sociodemographic marker rather than as a causal risk factor. Previous research has shown that marital status and partner drinking may be related to alcohol-related outcomes, although the direction and magnitude of these relationships depend strongly on social and interpersonal context (15, 16). Partner drinking, marital functioning, relationship satisfaction, and other interpersonal processes may provide relevant contexts for further investigation (15, 16).

The binary classification used in the present analysis combined marriage and free union into a single stable-partnership category, potentially obscuring heterogeneity between different partnership arrangements. Moreover, household organization and partner-level drinking patterns were not directly measured. Qualitative or mixed-methods research would therefore be better suited to examining how partnership, household roles, and family dynamics relate to alcohol-related concerns in this community.

Educational attainment showed descriptive differences across categories, although estimates for technical and university education were imprecise because of the small numbers of records. In the sensitivity model incorporating education, the adjusted prevalence ratio per increasing educational category was 0.91, with a confidence interval spanning the null. These findings provide limited evidence for an independent association with educational attainment. Nevertheless, previous research suggests that educational attainment and educational trajectories may intersect with alcohol use and social and emotional well-being in Indigenous populations (17, 18). Future studies should therefore examine educational experiences more comprehensively rather than relying solely on highest educational attainment.

The meaning of the primary outcome remains central to interpretation. The census item identified whether alcohol was selected as the “frequent problem” within the health and development component of the census. It did not measure individual consumption, drinking frequency or quantity, episodic heavy drinking, hazardous use, alcohol use disorder, or age at initiation. Consequently, the observed 64.8% should not be interpreted as the prevalence of individual alcohol consumption or alcohol use disorder.

Previous research has documented substantial alcohol-related concerns among Indigenous populations while emphasizing that these outcomes must be interpreted within their specific historical, social, and cultural contexts (3, 10, 19). Studies using instruments such as the Alcohol Use Disorders Identification Test have reported alcohol-related outcomes among Indigenous populations in Colombia and the Brazilian Amazon (4, 8). However, direct numerical comparison with the present estimate is inappropriate because those studies measured individual alcohol consumption or problem drinking, whereas the present census item recorded whether alcohol was identified as a frequent problem.

This distinction is particularly important because deficit-based interpretations can reinforce inappropriate generalizations about Indigenous populations. Contemporary literature instead emphasizes colonization, dispossession, historical and intergenerational trauma, structural inequalities, and commercial influences as relevant contexts for alcohol-related harms (10, 19, 20). These factors should be understood as broader social conditions rather than as characteristics inherent to Indigenous identity.

Nuevo Amanecer is located within the urban and peri-urban context of Shell, a setting described as important for the territorial, social, cultural, and economic dynamics of Waorani families (21). The present study cannot determine whether urbanization or broader processes of social change contributed to the reporting pattern observed in the census. Nevertheless, this context is relevant when interpreting alcohol as a frequently identified concern within this particular settlement.

The census did not measure functional, family, economic, educational, or health consequences directly attributable to alcohol. Therefore, the present findings cannot quantify the impact of alcohol-related concerns on everyday life in Nuevo Amanecer. Understanding these dimensions will require community-led qualitative and mixed-methods research focused on locally defined consequences, protective factors, and priorities.

Research and public health responses should recognize Indigenous leadership and community participation. Family-based approaches have been evaluated as one potential component of alcohol-harm reduction (7), while Indigenous-led regulatory interventions illustrate the importance of community ownership and consultation in the development of alcohol-related policies and responses (22). These principles are particularly relevant when findings arise from a small and identifiable Indigenous community.

Several methodological features strengthen the study. The analysis reconstructed variables directly from the original coded census source, retained non-classifiable values rather than imposing unsupported recoding, and reported the complete distribution of the frequent-problem item. The statistical approach used prevalence ratios for a common binary outcome and distinguished crude from adjusted estimates. Sensitivity analyses restricted to adults were particularly informative because they showed that the apparent age and partnership associations in the full model were less stable than the association with sex. Additional robustness analyses are presented in Supplementary Table S2.

Evidence from culturally adapted substance-use interventions in other populations indicates that language, context, content, and sociocultural fit are important dimensions of adaptation (23, 24). Although these studies do not establish the validity of alcohol-assessment instruments for Waorani populations, they reinforce the need to evaluate cultural and linguistic appropriateness before applying measures developed in other settings.

Several limitations should be acknowledged. First, the cross-sectional design does not establish temporality or causality. Second, the primary outcome was based on a single census item rather than on a validated measure of individual alcohol exposure or alcohol-related disorder. The study therefore cannot distinguish abstinence, occasional drinking, hazardous use, dependence, or specific alcohol-attributable harms.

Third, although the census procedures established a minimum age of 15 years for direct response and required parental authorization and informed assent for minors aged 15–17 years who participated directly, the analytical database did not retain respondent identity or direct-versus-proxy reporting status for individual records. Consequently, it was not possible to determine retrospectively which eligible adolescents responded directly and which records were completed by the household head. Because the frequent-problem item relied on respondent reporting, reporting bias, social desirability, differential interpretation of the item, and outcome misclassification cannot be excluded. These factors may have contributed to the contrasts observed between minors and adults.

Fourth, a unique household identifier was not available in the analytical dataset. Consequently, the number of unique households and average household size could not be reliably reconstructed. Individuals within the same household may share exposures, perceptions, and reporting sources, but potential within-household correlation could not be explicitly modeled. HC3 robust variance estimation addresses misspecification of the conventional Poisson variance but does not account for unmeasured household clustering.

Fifth, the modest number of census records resulted in sparse observations within some categories, particularly higher educational levels and some marital-status groups. This limited statistical precision and the complexity of multivariable adjustment. Relevant variables such as occupation, household socioeconomic conditions, drinking context, mental health, frequency and quantity of alcohol use, and access to alcohol were also unavailable, leaving the possibility of residual confounding.

Finally, this study represents a single Waorani settlement in an urban/peri-urban context. The findings should not be generalized automatically to other Waorani communities or other Indigenous populations. Communities experiencing different territorial, geographic, socioeconomic, or service-access conditions may present different patterns.

From a public health perspective, the frequent identification of alcohol in the census supports further community-led assessment rather than clinical labeling of individuals. Indigenous-specific evidence supports the importance of culturally tailored, family-based, community-led, and locally delivered approaches to alcohol and substance-use prevention and response (5, 7, 22, 25, 26). Broader evidence on culturally sensitive prevention and alcohol-related harm in rural and remote settings further suggests that interventions should be adapted to the sociocultural and geographic context in which they are implemented (27, 28).

For Nuevo Amanecer, future research should incorporate alcohol-specific measures capable of distinguishing alcohol consumption, hazardous drinking, alcohol-related harms, and alcohol use disorder, with appropriate cultural and linguistic adaptation and validation before their use in the Waorani context. Future data systems should also retain respondent identity, direct-versus-proxy reporting status, household identifiers, frequency and quantity of alcohol use, episodic heavy drinking, and alcohol-related consequences.

Longitudinal studies would help clarify temporal relationships and identify both risk and protective factors. Qualitative and mixed-methods research should explore local meanings of alcohol, household dynamics, gender roles, youth and adult transitions, and community-defined priorities. Ultimately, future interventions should be developed and evaluated in collaboration with Waorani leadership and community members, with attention to cultural safety, acceptability, sustainability, and locally defined outcomes (5, 22, 25, 26).

## Conclusion

In this community-based cross-sectional census of the Nuevo Amanecer Waorani settlement, alcohol was selected as the frequent problem in nearly two-thirds of census records. Male sex was the most consistent sociodemographic correlate across primary and adult-only analyses, whereas the associations with age and partnership status were more sensitive to the analytical population and should be interpreted cautiously. These findings should not be considered estimates of individual alcohol consumption, hazardous drinking, or alcohol use disorder, because the census item captured whether alcohol was identified as a frequent problem rather than individual drinking behavior. Future research should incorporate culturally appropriate validated measures, respondent and household identifiers, and longitudinal or mixed-methods approaches developed in collaboration with Waorani leadership to better characterize alcohol-related concerns and guide community-informed prevention strategies.

## Data Availability

The original contributions presented in the study are included in the article/Supplementary material, further inquiries can be directed to the corresponding authors.
