# Supplementary material for: Alcohol as a reported frequent problem and sociodemographic correlates in a Waorani community in the Ecuadorian Amazon: a community-based cross-sectional census study
Source: Front Public Health. 2026 Oct 19;14:1898589. doi: 10.3389/fpubh.2026.1898589 (PMC13724604; doi:10.3389/fpubh.2026.1898589)
Supplement: Supplementary file 1 [file Table_1.docx]

**Supplementary Material**

**Supplementary File S1. Community census form used in Nuevo Amanecer**

**Original form title: “CENSO ASENTAMIENTO NUEVO AMANECER FAMILIAS WAORANI”**

**Transcription note.** This supplement reproduces the content and response options visible in the original community census form in a structured format for readability. Original Spanish wording is retained. English descriptors are provided only to facilitate interpretation by readers.

**Primary outcome item used in the present analysis**

| **Component** | **Content** |
| --- | --- |
| **Original item as printed** | Problemática frecuente: 1. Alcohol 2. Droga 3. Sectas 4. Otra ¿Cuál? |
| **English descriptor** | Frequent problem: 1. Alcohol; 2. Drugs; 3. Sects; 4. Other, specify. |
| **Analytical coding note** | The source analytical database additionally contained code 0 = “Ninguno” (none). This code is present in the database but is not listed among the visible printed response options on the source form. |

**A. Identification and visit information**

| **Original field (Spanish)** | **English descriptor** | **Response / field type** |
| --- | --- | --- |
| Comunidad | Community | Nuevo Aamanecer [as printed in the community field] |
| Zona | Zone | Shell |
| Sector | Sector | WAORANI |
| # Casa | House number | Open field |
| Equipo que censa | Census team | Open field |
| Domicilio o datos de referencia | Address or reference information | Open field |
| Número de familias que viven en la casa | Number of families living in the household | Open field |
| Compromiso de la visita. Marcar con “x” | Visit commitment; mark with X | 1. Visita; 2. Apoyo comunitario; 3. Otro |

**B. Instructions printed on the census form**

- “NO HAGAS UNA PAUSA PARA LLENAR EL CENSO”.
- Recuerda que con esta información lograremos “resolver problemas y detectar oportunidades”.
- Levanta el censo una sola vez. Si no obtienes los datos importantes, regresa por ellos. No entregues censos incompletos.
- Llenar el censo con letra legible.
- No olvides ubicar la casa por zona, sector, número de casa y el mejor dato de referencia posible (breve).
- Si encuentras alguna casa no marcada en el mapa, no olvides visitarla, dibujarla en tu mapa, censarla y reportarla para que se actualice.

**C. Individual, household, and community variables**

| **Domain** | **Original item / field (Spanish)** | **English descriptor** | **Response options / coding shown on form** |
| --- | --- | --- | --- |
| Identificación | Nombre, paterno, materno | Full name / paternal surname / maternal surname | Open text field |
| Datos generales | Parentesco | Relationship to household head | 1. Padre; 2. Madre; 3. Hijo; 4. Nieto; 5. Hermano; 6. Tío; 7. Sobrino; 8. Yerno; 9. Nuera |
| Datos generales | Edad | Age | Numeric / open field |
| Datos generales | Sexo (m, f) | Sex | M; F |
| Datos generales | Lee y escribe (sí/no) | Literacy | Sí; No |
| Religión | Religión | Religion | 1. Católica; 2. Evangélica; 3. Testigo de Jehová; 4. Mormona; 5. Cristianos; 6. Otra, ¿cuál? |
| Datos generales | Estado civil | Marital status | 1. Casado; 2. Unión libre; 3. Divorciado; 4. Madre soltera; 5. Soltero |
| Territorio | Territorio | Territory | Open field |
| Vida parroquial | Bautizo (sí/no) | Baptism | Sí; No |
| Hogar / servicios | Agua (sí/no) | Water service | Sí; No |
| Hogar / servicios | Luz (sí/no) | Electricity | Sí; No |
| Tecnología | Tecnología | Available communication / media technology | 1. Celular; 2. Convencional; 3. TV satelital; 4. Internet; 5. Ninguno |
| Estudios | Estudios | Educational attainment | 1. Educación básica; 2. Bachillerato; 3. Tecnología; 4. Universidad; 5. Otro |
| Actividad física | Practica deporte | Sports participation | Open / yes-no field as printed |
| Participación comunitaria | Participa en algún proyecto | Participation in a project / organization | 1. ANWAE; 2. NAWE; 3. CONCONAWEP |
| Salud y desarrollo | Enfermedad crónica | Chronic disease | 1. Respiratorias; 2. Gastro; 3. Discapacidad; 4. Otra |
| Salud y desarrollo | Problemática frecuente | Frequent problem | 1. Alcohol; 2. Droga; 3. Sectas; 4. Otra, ¿cuál? |
| Salud | Salud | Self-rated / recorded health status | 1. Buena; 2. Regular; 3. Mala |
| Origen | Comunidad donde nació | Community of birth | Open text field |
| Hogar | Casa | Housing characteristics / status | 1. Madera; 2. Cemento; 3. Mixto; 4. Escritura; 5. Sin papeles de la casa? |
| Ocupación | Oficio | Occupation / trade | Open text field |
| Participación | Interesado en colaborar sí/no. ¿cómo? | Interest in collaborating | Sí/No; open field for “¿Cómo?” |

**Source-form note.** This is a structured transcription of the original census form supplied for manuscript revision. It is intended to make the instrument, item wording, and response options transparent. It does not add psychometric properties or response categories that are not visible in the source form, except where the analytical coding note explicitly documents database code 0 = “Ninguno”.

**Supplementary Table S2. Model diagnostics and robustness analyses**

**Analytical note.** The primary complete-case model included sex, age group, and partnership status and was estimated using a modified Poisson generalized linear model with log link. HC3 robust sandwich variance was used for the primary analysis. The tables below summarize diagnostic and robustness checks that were intentionally kept out of the main Methods section for concision.

**S2A. Primary model diagnostics**

| **Diagnostic** | **Result** |
| --- | --- |
| Observations | 123 |
| Events / non-events | 80 / 43 |
| Model parameters, including intercept | 7 |
| Events per parameter | 11.43 |
| Convergence | Yes |
| Residual degrees of freedom | 116 |
| Pearson chi-square / residual df | 0.363 |
| Deviance / residual df | 0.443 |
| Primary variance estimator | HC3 robust sandwich |
| Adjusted GVIF: sex | 1.045 |
| Adjusted GVIF: age group | 1.087 |
| Adjusted GVIF: partnership status | 1.349 |
| Maximum Cook’s distance | 0.0144 |
| Household-clustered variance | Not estimable because a unique household identifier was unavailable |

**S2B. Sensitivity to robust covariance specification**

| **Contrast** | **PR** | **HC0 95% CI** | **HC1 95% CI** | **HC3 95% CI** |
| --- | --- | --- | --- | --- |
| Male vs female | 1.31 | 1.04–1.66 | 1.04–1.67 | 1.03–1.67 |
| Age 18–24 vs <18 years | 2.64 | 1.61–4.33 | 1.59–4.40 | 1.58–4.42 |
| Age 25–34 vs <18 years | 2.64 | 1.60–4.33 | 1.58–4.40 | 1.57–4.42 |
| Age 35–44 vs <18 years | 1.72 | 0.96–3.08 | 0.94–3.13 | 0.94–3.16 |
| Age ≥45 vs <18 years | 2.11 | 1.20–3.72 | 1.18–3.78 | 1.16–3.83 |
| Stable partner vs no stable partner | 1.30 | 1.02–1.65 | 1.02–1.66 | 1.004–1.677 |

**Note.** Point estimates are identical across HC0, HC1, and HC3 because only the covariance estimator changes. The primary analysis used HC3. Values are rounded; the stable-partnership HC3 confidence interval is shown to three decimals because its lower limit is close to 1.00.

**S2C. Leave-one-out stability of primary adjusted prevalence ratios**

| **Contrast** | **Successful refits** | **Median PR** | **PR range across refits** |
| --- | --- | --- | --- |
| Male vs female | 123/123 | 1.314 | 1.278–1.348 |
| Age 18–24 vs <18 years | 123/123 | 2.640 | 2.568–2.861 |
| Age 25–34 vs <18 years | 123/123 | 2.632 | 2.554–2.868 |
| Age 35–44 vs <18 years | 123/123 | 1.713 | 1.580–1.921 |
| Age ≥45 vs <18 years | 123/123 | 2.100 | 1.983–2.350 |
| Stable partner vs no stable partner | 123/123 | 1.298 | 1.222–1.371 |

**Note.** Each observation was omitted once and the primary model was refitted. All 123 leave-one-out models converged. The table summarizes stability of the adjusted prevalence-ratio point estimates; it is not intended as a separate inferential analysis.

**S2D. Sensitivity analysis additionally adjusting for educational attainment**

| **Characteristic** | **aPR** | **95% CI** | **p value** |
| --- | --- | --- | --- |
| Male vs female | 1.34 | 1.05–1.71 | 0.019 |
| Age 18–24 vs <18 years | 2.66 | 1.60–4.43 | <0.001 |
| Age 25–34 vs <18 years | 2.65 | 1.59–4.41 | <0.001 |
| Age 35–44 vs <18 years | 1.73 | 0.95–3.15 | 0.075 |
| Age ≥45 vs <18 years | 2.19 | 1.22–3.92 | 0.008 |
| Stable partner vs no stable partner | 1.35 | 1.02–1.77 | 0.033 |
| Education, per ordered category | 0.91 | 0.80–1.04 | 0.185 |

**Note.** The education sensitivity model used only documented ordered education categories. Records with an undocumented education code were excluded rather than reassigned to an unsupported category. Education was modeled ordinally for this sensitivity analysis.
